# Congenital Glaucoma: a Novel Ocular Manifestation of Hajdu-Cheney Syndrome

**DOI:** 10.1155/2018/2508345

**Published:** 2018-10-21

**Authors:** L. Swan, G. Gole, V. Sabesan, J. Cardinal, D. Coman

**Affiliations:** ^1^Department of Paediatrics, The Wesley Hospital, Brisbane, Australia; ^2^Department of Ophthalmology, The Lady Cilento Children's Hospital, Brisbane, Australia; ^3^The School of Medicine, University of Queensland, Brisbane, Australia; ^4^Department of Paediatrics, The Townsville General Hospital, Townsville, Australia; ^5^Cardinal Bioresearch, Brisbane, Australia; ^6^Department of Metabolic Medicine, The Lady Cilento Children's Hospital, Brisbane, Australia; ^7^The School of Medicine, Griffith University, Gold Coast, Australia

## Abstract

Hajdu-Cheney Syndrome (HSC) is a rare multisystem disease in which the phenotype involves acro-osteolysis, severe osteoporosis, short stature, wormian bones, facial dysmorphism, central neurological abnormalities, cardiovascular defects, and polycystic kidneys. We describe an infant with severe manifestations of HCS in whom congenital glaucoma was a significant early feature, which has not been reported to date. HCS cases reported to date have involved truncating mutations in exon 34 of* NOTCH2* upstream the PEST domain that lead to the development of a truncated and stable NOTCH2 protein which upregluates notch signaling. We describe a hitherto undescribed missense mutation that is predicted to be pathogenic, with functional characterization remaining to be performed. Serpentine fibula-polycystic kidney syndrome (SFPKS) is allelic to HCS and commonly associated with missense* NOTCH2 *mutations. Our patient provides new ophthalmological manifestations of HCS and provides insight into the potential role of notch signaling in the anterior chamber development.

## 1. Introduction

Hajdu-Cheney Syndrome (HCS)(OMIM#102500) is a rare autosomal dominant disease due to gain of function mutations in the* NOTCH2* (OMIM #600275) gene [1–3]. HCS is a multisystem disorder characterized by acro-osteolysis of distal phalanges, wormian bones, severe osteoporosis with fractures, short stature, cardiac malformations, central nervous system involvement, dental anomalies, cystic renal disease, and craniofacial dysmorphism [4, 5].

Ophthalmic manifestations have been reported in HCS, though they may not have been directly related to the syndrome, and have included severe myopia [6, 7], nystagmus [8, 9], optic disc swelling [9], optic nerve meningoceles [10], cataracts [7], and hypertensive retinopathy [11].

Herein we present a case of HCS who developed congenital glaucoma in the first week of life. Other features included dysmorphism, macrocephaly, wormian bones, brachydactyly, and communicating hydrocephalus and a thin corpus callosum. To the best of our knowledge this is the first report of congenital glaucoma in HCS. We postulate that our patient with a HCS clinical phenotype and a novel de novo missense mutation in the PEST domain of* NOTCH2* expand the genotype-phenotype spectrum between HCS and its allelic condition Serpentine fibula-polycystic kidney syndrome (SFPKS) (OMIM#102500)

## 2. Case Report

The male child was born at 36+6 weeks to nonconsanguineous parents. Antenatal ultrasound scan identified severe polyhydramnios and mild ventriculomegaly, with an emergency caesarean occurring secondary to a head circumference measuring large for dates. Immediately postbirth he required special care nursery admission for blood glucose monitoring due to maternal gestational diabetes. At birth, his weight was 2612g, length 46.5cm, and head circumference 35.4cm.

At 10 days of age, he was noted to have a cloudy right cornea. His intraocular pressures at this time were 22 mmHg right eye and 16 mmHg left eye [10–18]. His horizontal corneal diameters were 12.5 mm right eye and 10.5 mm left eye. The right axial length was 18.6 mm right eye and 17.0 mm left eye. The cup/disc ratio was 0.7 right eye and 0.5 left eye. Thus, a diagnosis of right sided congenital glaucoma was made. He was commenced on topical B blockers and underwent an uneventful right trabeculotomy at 2 weeks of age. He subsequently required a topical prostaglandin analogue (latanaprost) for pressure control in the right eye.

**Figure 1:**
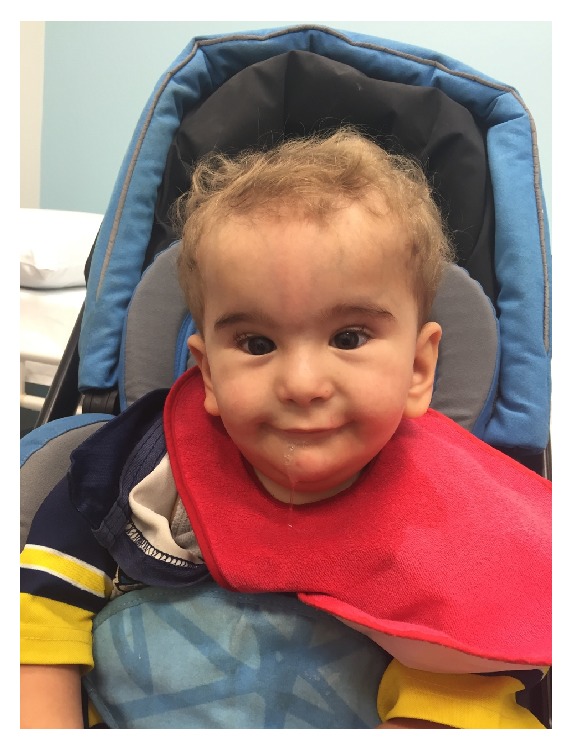
Facial image of the child at 3 years of age demonstrating dysmorphic features consisting of macrocephaly, coarse facial features, flat midface, micrognathia, coarse hair, telecanthus, smooth philtrum, low set ears, synophrys, and long eye lashes.

In addition to congenital glaucoma, the child also exhibited dysmorphic features consisting of macrocephaly, coarse facial features, flat midface, micrognathia, coarse hair, open cranial sutures, telecanthus, smooth philtrum, low set ears, synophrys, and long eye lashes (see Figure 1). He had small hands and feet with tapered, short fingers with fingers 3-4 showing syndactyly as well as syndactyly of his 2-3 toes. Both of his palms exhibited single palmar creases. He had cryptorchidism which required surgical management. He has significant global developmental delay, and central hypotonia. PEG insertion was required for supplemental feeding secondary to the significant hypotonia.

An MRI at age 16 months showed a communicating hydrocephalus (with prominent subarachnoid spaces), progressive dilation of the ventricles, and thinning of the corpus callosum. Skull x-ray showed wormian bones but there was no platybasia. There was a significant reduction of the femoral neck angle bilaterally. He remains below the 1^st^ centile for age for weight and height, with a current height of 94.5cm, weight 15.3kg, and greater than the 95^th^ centile for head circumference of 53cm (at current age of 4 years). A renal ultrasound at birth showed no significant deformity. Microscopic examination of hair follicles showed tichorrhexis nodosa with fibre separation and fracture. The hair samples also showed variation, with some hairs showing one half of an area of trichorrhexis at one end and trichoptilosis with widely splayed fibres and simple knots. Chromosonal SNP array, urine metabolic studies, copper studies, cholesterol studies, 7/8 dehydrocholesterol, creatine kinase, transferrin isoforms, APOC3, and very long chain fatty acids were found to be normal.

## 3. Whole Exome Sequencing

A singleton exome and subsequent parental and patient sanger sequencing were performed in the Macrogen laboratories (http://www.macrogen.com/eng/). After enrichment of all the coding and flanking intronic regions of the genes mentioned above, sequencing analysis was performed using an Illumina HiSeq platform. 97.7% of targeted regions achieved x100 coverage and 99.7% achieved x10 coverage. Only clinically relevant sequence variations with an allele frequency <0.1% were considered. A missense variant of uncertain significance was detected* in NOTCH2* 7066G>A (Ala2317Thr), at position 120,458,279 bp in exon 34. The mutation was confirmed as* de novo* via Sanger sequencing. The variant has not been previously reported on dbSNP. Minor/alternative allele frequencies are not reported in the 1000 genome or the NHLBI GO Exome Sequencing. This variant overlaps with evolutionary constrained element, detected using SiPhy-*ω* and SiPhy-*π* statistics. The conservation across 28 species is described with PhyloP (score 0.64). The variant is within the PEST domain region.

## 4. Discussion

HCS is characterized by abnormal bone turnover with variable severity and associated craniofacial features and cardio-renal defects [1, 12]. The identified causative heterozygous truncating mutation for HCS has been found to be within the* NOTCH2* domain, located on chromosome 1p12 [1, 12]. Prior to the molecular aetiology of HCS being discovered, Brennan et al. described 10 key clinical manifestations of HCS [5]. These included (1) acroosteolysis, (2) wormian bones, (3) platypasia, (4) premature loss of teeth, (5) micrognathia, (6) coarse face, (7) coarse hair, (8) midface flattening, (9) short stature (<5%), and (10) a positive family history [5]. With paediatric cases like our child, in whom there was no supportive family history, a diagnostic of at least 4 criteria were required to support a clinical diagnosis [5]. Our patient currently displays 6 features, i.e., wormian bones, micrognathia, coarse facial features, coarse hair, flattened midface, and short stature.

The majority of mutations identified to date have been found to affect the Proline-glutamic acid-serine-threonine-rich (PEST) domain at the C-terminus of the receptor via frameshift or nonsense mutations [12]. This domain has an essential role in proteasomal degradation of the intracellular Notch domain [1, 2, 12]. Pathogenic* NOTCH2 *mutations are able to avoid nonsense mediated mRNA decay causing gain of function defects, resulting in increased* NOTCH2* signalling [1, 2, 12]. This domain has an essential role in proteasomal degradation of the intracellular Notch domain [12]. Pathogenic* NOTCH2 *mutations are able to avoid nonsense mediated mRNA decay causing gain of function defects [12]. The sequence variant identified in this report is a de novo missense mutation adding an inappropriate threonine to the PEST domain. The PEST sequence is very well conserved for all NOTCH proteins. Our patient has a missense variant in* NOTCH2* 7066G>A (Ala2317Thr), at position 120,458,279 bp in exon 34., within the PEST domain. The variant has not been previously recorded previously and is predicted to be pathogenic. We hypothesise that the addition of an extra threonine may interrupt proteasomal degradation of NOTCH2. However, functional studies are needed to confirm this to be the case.

Serpentine fibula-polycystic kidney syndrome (SFPKS) is allelic to HCS as its also due to* NOTCH2 *mutations [13], especially missense mutations [2, 11, 14]. There are phenotypic similarities between HCS and SFPKS including bone abnormalities, specifically, wormian bones and serpentine fibulas, craniofacial abnormalities, and cystic kidneys [11, 13, 14]. Despite the similarities between the disorders, congenital glaucoma has not been described in the literature in either SFPKS or HCS. We postulate that our patient with a HCS clinical phenotype and a novel* de novo* missense mutation in the PEST domain of* NOTCH2 *expand the gen0type-phenotype spectrum between HCS and SFPKS.

HCS is characterized by abnormal bone turnover with variable severity and associated craniofacial features and cardio-renal defects [12] The NOTCH signalling pathway plays a key role in the commitment of pluripotent osteoblastic precursors as well as the suppression of differentiation of osteoblasts [1, 12] and is a crucial mediator in osteoblast cell fate [15]. The phenotypic findings in HCS that particularly relate to abnormal bone turnover are acro-osteolysis and wormian bones in the skull sutures. Acro-osteolysis is the progressive shortening of the digits secondary to bone resorption of the phalanges with underlying additional bone abnormalities [3]. The wormian bone formation is likely secondary to disruption of the normal embryonic role of* NOTCH2 *in the cranial skeleton [15].

Congenital glaucoma usually arises from abnormal embryological development of the anterior chamber angle. Anterior segment dysgenesis occurs when the structures of the anterior segment of the eye are affected, such as the cornea, iris and lens, anterior chamber and posterior chamber, trabecular meshwork, and Schlemm's canal [16]. Aqueous humour is produced by the ciliary body, passes from the posterior chamber via the pupil into the anterior chamber, and then drains through the trabecular meshwork into Schlemm's canal [16]. Any dysregulation in this drainage process or flow leads to increased intraocular pressure and a subsequently increased for developing glaucoma [16]. Zhou et al. demonstrated in murine models that* Notch2* was required for morphogenesis and cell signalling of the ciliary body [17].* NOTCH2 *gene mutation have been explored as candidates for primary open angle glaucoma due to its expression in the anterior segment of the eye [18].

HCS is a rare genetic disease; our patient expands the clinical phenotype to include congenital glaucoma. We propose that all HCS patients should be screened for glaucoma.
